# Editorial: Infectious diseases and hematology: diagnosis and management

**DOI:** 10.3389/fmed.2024.1385874

**Published:** 2024-03-14

**Authors:** Tomás José González-López, Isidro Jarque, Carmine Siniscalchi, Pierpaolo Di Micco

**Affiliations:** ^1^Department of Hematology, Hospital Universitario de Burgos, Burgos, Spain; ^2^Department of Hematology, Hospital Universitario y Politécnico La Fe, Valencia, Spain; ^3^Department of Internal and Emergency Medicine, Parma University Hospital, Parma, Italy; ^4^AFO MEDICA P.O. Santa Maria delle Grazie, ASL NA2 Nord, Naples, Italy

**Keywords:** infections, hematology, diagnosis, management, vaccination

It is crucial to understand the immune status of hematological patients, as it significantly influences the severity and types of infections to which they are susceptible. Patients with neutropenia are at high risk of severe bacterial infections, and prolonged neutropenia increases the risk of fungal infections. Moreover, impaired T-cell function increases the likelihood of fungal and viral infections. Knowledge of local resistance patterns is essential to guide empirical antimicrobial therapies (1).

Regarding management, it is important to focus on prevention, diagnosis, and treatment. Thus, vaccines and anti-infective prophylaxis have improved survival rates for these patients. Having the best possible strategies for managing infections is fundamental for lowering the chances of illness and death for those who do get sick (2).

This compendium points to several issues. Thus, diverse novel aspects of the management and/or treatment of various infections, the validation of predictive risk models for infections in hematology, the COVID-19 behavior in certain populations, the optimal assessment of the response to some COVID-19 vaccinations, and atypical responses to treatments, e.g. antifungals such as voriconazole, are included here. This volume is completed by two articles focusing on other related aspects of hematology.

Pando-Caciano et al. address the management of refractory cytomegalovirus (CMV) infections in the context of pediatric haematopoietic stem cell transplantation (HSCT) highlighting the use of highly sensitive genetic tools to investigate virus resistance in a broader genome-wide range. This is the first reported case in Latin America of refractory CMV infection in a pediatric HSCT recipient without evidence of clinical symptoms and CMV genetic resistance.

Ge et al. report an intra-abdominal Mycobacterium syngnathidarum infection in an immunocompetent patient. This is the first report of Mycobacterium syngnathidarum infection in humans. This mycobacterium was detected by whole genome sequencing (WGS). Therefore, the authors suggest that WGS can serve as a high-resolution assay for the diagnosis of different subtypes of mycobacterial infection.

Cao et al. report a case of haemophagocytic lymphohistiocytosis (HLH) with detection of Rickettsia DNA in blood that met the diagnostic criteria of the Histiocyte Society's HLH-2004 guidelines. The patient was treated with antibiotics, glucocorticoid therapy and continuous renal replacement therapy (CRRT) temporarily improving his condition. However, the patient died 2 years later due to chronic renal failure caused by septic shock.

Zhou et al. address the risk of nosocomial infections with multi-drug resistant organisms (MDRO) in neonatal intensive care units (NICUs). Authors conducted a multicenter observational study at NICUs of two tertiary children's hospitals in China. This study is the first to construct a predictive risk model (PRM) for nosocomial infections with MDRO in NICUs.

Li et al. validate another PRM to establish the prognosis of hospitalized patients with severe fever with thrombocytopenia syndrome (SFTS) before reaching the critical illness stage and compare the predictive ability of groups with and without viral load. This PRM represents a convenient tool for early identification of critically ill patients in order to initiate better personalized treatment in time.

Huang et al. show their prediction of the risk of cytopenias during hospitalization in HIV/AIDS patients. The authors used artificial intelligence (five machine learning prediction models) to analyse the data and concluded hypoproteinaemia and cancer were the most important predictors in this context.

Ali et al. address the issue of COVID-19 in patients with chronic myeloid leukemia (CML). With respect to the controversy over whether CML patients have better outcomes than normal people or not, this review evaluates the outcome of CML patients with COVID-19 and attempts to address the missing area of knowledge.

Ghaffari et al. investigated human platelet antigen (HPA)-1 and HPA-3 (GPIIb/IIIa), HPA-2 (GPIb/IX), HPA-4 (GPIIIa), HPA-5 (GPIa/IIa) and HPA-15 (CD109) polymorphisms in 86 COVID-19-infected patients with thrombocytopenia and 136 COVID-19-infected patients without thrombocytopenia. The authors present here the first evidence suggesting the distinct association of specific combinations of HPA genotypes with thrombocytopenia in COVID-19 infected patients. They recommend evaluating the role of HPA polymorphisms as risk factors for thrombocytopenia in different COVID-19 populations.

Xie et al. present a rare case of a patient with systemic lupus erythematosus with a fungal infection who developed MDS-like adverse reactions after treatment with voriconazole. Because the patient's sputum culture showed Candida albicans infection, oral voriconazole was prescribed. After the use of voriconazole, drug-related adverse reactions such as visual disturbances, nausea, vomiting and others, as well as a gradual increase in serum creatinine and oliguria appeared. In addition, there was a decrease in peripheral blood cells, and MDS-like changes were observed in the bone marrow by bone marrow biopsy. After stopping voriconazole, drug-related adverse symptoms disappeared, while cytopenias and MDS changes improved significantly.

Szabó et al. show their results with 49 patients with hematological diseases (HD) and 46 healthy controls (HCs) enrolled to receive a full two-dose vaccination with three different SARS-CoV-2 vaccines (BNT162b2, or AZD1222, or BBIBP-CorV).

Although, humoral immune activity against SARS-CoV-2 can be highly evoked by the BNT162b2 mRNA-based vaccination compared to the other two, the authors demonstrate a significant weaker overall response to the vaccines in the immunologically deficient HD population against HCs, regardless of vaccine type.

Xu et al. investigate the feasibility and accuracy of liver iron concentration (LIC) quantification in thalassaemia (TM) patients using 1.5T and 3T T2^*^ magnetic resonance imaging (MRI).

Li et al. confirm here that, in pediatric patients with hemophilia A, the US scoring system correlated well with the Hemophilia Joint Health Score (HJHS 2.1) for global and individual joint assessments, with excellent correlations for elbows, substantial correlations for knees and moderate correlations for ankles.

The articles of this Research Topic describe different and often insufficiently known aspects of the diagnosis and management of various infectious diseases in our hematological patients. Further studies following the results observed here will undoubtedly contribute to a better understanding of these issues.

## Author contributions

TJG-L: Conceptualization, Formal analysis, Methodology, Supervision, Validation, Visualization, Writing – original draft, Writing – review & editing. IJ: Writing – original draft, Writing – review & editing. CS: Writing – original draft, Writing – review & editing. PD: Writing – original draft, Writing – review & editing.
